# Comparison of zero heat flux and double sensor thermometers during spinal anaesthesia: a prospective observational study

**DOI:** 10.1007/s10877-021-00799-6

**Published:** 2022-01-03

**Authors:** Sirkka-Liisa Lauronen, Maija-Liisa Kalliomäki, Jarkko Kalliovalkama, Antti Aho, Heini Huhtala, Arvi M. Yli-Hankala, Marja-Tellervo Mäkinen

**Affiliations:** 1 Department of Anaesthesia, Tampere University Hospital, Elämänaukio 2, POB 2000, 33521 Tampere, Finland; 2 Coxa Hospital for Joint Replacement, Tampere, Finland; 3 Faculty of Social Sciences, Tampere University, Tampere, Finland; 4 Faculty of Medicine and Health Technology, Tampere University, Tampere, Finland; 5 Department of Anaesthesiology, Intensive Care and Pain Medicine, University of Helsinki, and Helsinki University Hospital, Helsinki, Finland

**Keywords:** Non-invasive core temperature measurement, Zero heat flux, Double sensor, Spinal anaesthesia

## Abstract

Because of the difficulties involved in the invasive monitoring of conscious patients, core temperature monitoring is frequently neglected during neuraxial anaesthesia. Zero heat flux (ZHF) and double sensor (DS) are non-invasive methods that measure core temperature from the forehead skin. Here, we compare these methods in patients under spinal anaesthesia. Sixty patients scheduled for elective unilateral knee arthroplasty were recruited and divided into two groups. Of these, thirty patients were fitted with bilateral ZHF sensors (ZHF group), and thirty patients were fitted with both a ZHF sensor and a DS sensor (DS group). Temperatures were saved at 5-min intervals from the beginning of prewarming up to one hour postoperatively. Bland–Altman analysis for repeated measurements was performed and a proportion of differences within 0.5 °C was calculated as well as Lin`s concordance correlation coefficient (LCCC). A total of 1261 and 1129 measurement pairs were obtained. The mean difference between ZHF sensors was 0.05 °C with 95% limits of agreement − 0.36 to 0.47 °C, 99% of the readings were within 0.5 °C and LCCC was 0.88. The mean difference between ZHF and DS sensors was 0.33 °C with 95% limits of agreement − 0.55 to 1.21 °C, 66% of readings were within 0.5 °C and LCCC was 0.59. Bilaterally measured ZHF temperatures were almost identical. DS temperatures were mostly lower than ZHF temperatures. The mean difference between ZHF and DS temperatures increased when the core temperature decreased.

Trial registration: The study was registered in ClinicalTrials.gov on 13th May 2019, Code NCT03408197.

## Introduction

Body core temperature monitoring is often neglected during neuraxial (i.e., spinal or epidural) anaesthesia (NA) [1, 2] even though NA is known to predispose a patient to hypothermia [3] and resultant adverse effects [4–9]. Conscious patients under NA do not perceive temperature changes, and thus, although hypothermic, will not report feeling cold [10]. Vigilance by the anaesthesia team and adequate temperature measurement are therefore mandatory to ensure the detection and prevention of perioperative temperature disturbances.

Core temperature can be reliably measured from a pulmonary artery (PA) or other well perfused sites, such as the oesophagus, nasopharynx and tympanic membrane. The bladder and rectum are less reliable sites because they are poorly perfused. Because all these measurement sites are invasive, they are unsuitable for conscious patients. Moreover, the conventional sites used for the core temperature measurement of conscious patients (e.g., oral, axillar or infrared measurements from the tympanic membrane or temporal region of the head) typically lack the desired clinical accuracy and reliability [11].

In recent years, other core temperature measurement devices have been developed. In 1971 Fox and Solman demonstrated a non-invasive zero heat flux (ZHF) method for measuring core temperature from the intact skin surface [12, 13]. This technique was subsequently further developed in Japan [14, 15]. The ZHF system (3M™ BairHugger™ Temperature Monitoring System, previously 3M™SpotOn™, Arizant Healthcare, Eden Prairie, MN, USA) has been proven to be accurate and precise enough when compared with standard invasive core temperature measurements [16–19]. Another non-invasive core temperature measurement system, which incorporates the double-sensor (DS) technique, was released in 2006 (Tcore™ Temperature Monitoring System, Drägerwerk AG & Co, Lübeck, Germany). In noncardiac clinical studies, the DS method was estimated to be sufficient for routine clinical use [20, 21], whereas the agreement between DS and PA temperature measurements in cardiac surgical patients was less satisfactory [22, 23].

In previous studies, the ZHF and DS methods have been compared to standard core temperature measurement methods. To the best of our knowledge, however, no previous studies have compared the ZHF and DS methods or two bilateral ZHF sensors placed simultaneously on both sides of a patient`s forehead. Our study evaluates these two non-invasive methods perioperatively in patients undergoing unilateral total knee arthroplasty under spinal anaesthesia. We hypothesised that the core temperature measured on either side of the forehead is similar, regardless of the method used.

## Methods

Ethical approval for the study (ETL R17136) was provided by the Regional Ethics Committee of the Expert Responsibility Area of Tampere University Hospital, Tampere, Finland (Chairperson Prof Matti Korppi) on 3rd October 2017. This observational study was registered in ClinicalTrials.gov on 13th May 2019 (Code NCT03408197). All patients gave their written informed consent prior to their inclusion in the study.

Sixty adult patients scheduled for elective, primary unilateral total knee arthroplasty under spinal anaesthesia were enrolled in the study. Exclusion criteria were body mass index (BMI) < 25 or > 40, American Society of Anesthesiologist (ASA) class > 3, general anaesthesia (GA) or inability to give written consent.

### Protocol

Patients arrived at the hospital and were recruited to the study on the day of the surgery. Paracetamol 1 g and cetirizine 10 mg were used as premedication. Before surgery, patients were prewarmed in supine position for thirty minutes in the preoperative holding area. Either a forced-air warming (FAW) blanket (3M™BairHugger™, model 62200,) or self-warming blanket (Barrier® EasyWarm®, Mölnlycke Health Care AB, Gothenburg, Sweden) was placed longitudinally on a patient`s body and legs during prewarming. After prewarming, standard monitoring (non-invasive blood pressure, electrocardiogram and SpO_2_) was applied, intravenous access was opened, and spinal anaesthesia was induced with isobaric bupivacaine (Bicain Spinal 5 mg/ml, Orion Pharma, Espoo, Finland) in lateral position. After ensuring the complete motor and sensory block of the lower limb to be operated, patients were transferred to the operating room (OR). During surgery, patients received propofol sedation for their comfort, if desired, which was induced and maintained with target-controlled infusion (TCI, Asena™ PK, Alaris Medical Systems, Basingstoke, UK). Propofol was initially administered with Schnider model effect-site concentration set to 1.0 µg/ml and adjusted when needed. Patients were under light sedation (score -2), or moderate sedation (score -3) as measured with The Richmond Agitation–Sedation Scale. No other sedation or opiates were used intraoperatively. Active warming was continued intraoperatively. Both blankets were placed on the chest and arms during surgery. The head of the patient was left uncovered.

Patient characteristics and relevant perioperative data were saved on an information security data collection file. Personal data were processed in accordance with the European Union`s General Data Protection Regulation requirements.

### Temperature monitoring

The ZHF and DS temperature monitoring systems consist of a disposable sensor, which is attached to the forehead skin above the eyebrow, and a reusable control unit or an adapter. The ZHF sensor is 41 mm high, 41 mm wide and 5 mm thick; the DS sensor is 49 mm high, 58 mm wide and 5 mm thick. The control unit of the ZHF system is compatible with existing monitors, but it requires current to work. However, the battery-powered adapter of the DS system is only compatible with Dräger monitors.

The ZHF and DS systems are both based on vertical heat flow from deep tissue to the skin surface. According to the manufacturer, the ZHF sensor consists of two thermistors and a covering flex circuit, which are separated by insulating foam. The flex circuit regulates its temperature to create a zone of perfect insulation. Thus, heat loss to the environment is eliminated and core temperature can be measured from the skin surface [24]. The DS sensor consists of two thermistors separated by insulating foam and a cover. The DS system determines core temperature by using the formula: T_c_ = T_1_ + K_insul_/K_tis_ × (T_1_−T_2_). Core temperature (T_c_) is estimated from the measured temperatures at each point of the thermistors (T_1_ and T_2_)_,_ and the ratio of the thermal conduction coefficient of the insulating foam (K_insul_) to that of human tissue (K_tis_) [25].

All patients had a ZHF sensor (3M™ Bair Hugger™ Temperature Monitoring System) placed on the right side of the forehead as the reference core temperature (T_ZHF-R_). The study sensor, either a ZHF sensor (T_ZHF-L_; ZHF group) or a DS sensor (Tcore™ Temperature Monitoring System; T_DS_; DS group), was placed on the left side of the forehead.

Temperatures were measured preoperatively, in the OR and up to one hour postoperatively. Temperature monitoring was temporarily interrupted while the patient was transferred to the OR or the recovery room. Temperatures of the ZHF group were collected at 10-s intervals using S5Collect software (GE Healthcare Oy, Helsinki, Finland) and retrieved for statistical analysis at 5-min intervals. Temperatures of the DS group were saved on the data collection file at 1-min intervals from the beginning up to ten minutes, and thereafter at 5-min intervals.

### Statistical analysis

For each patient, temperature data consisted of multiple measurements taken pre-, intra- and postoperatively. Bland–Altman (BA) analysis was used to assess the agreement between the two temperatures obtained by either two ZHF sensors or by a ZHF and a DS sensor. Mean difference and 95% limits of agreement (LoA: ± 1.96 standard deviation (SD) around the mean difference) with 95% confidence intervals (CI) were calculated as described by Zou with multiple observations per patient [26]. BA analysis was computed separately for the pre-, intra- and postoperative data. A difference of ± 0.5 °C between two temperatures was determined to be clinically acceptable [27]. The percentage of measurement differences within 0.5 °C were counted. Lin`s concordance correlation coefficient (LCCC) for repeated measures with 95% CI was calculated.

Statistical analyses were performed using SPSS Version 25.0. (IBM Corp: Armonk, NY), and STATA (StataCorp. 2019. College Station, TX: StataCorp LLC). BA plot with 95% CIs were calculated using Microsoft Excel 2010 (Microsoft, Redmond, WA, USA). Continuous data are reported as median and quartiles (Q_1_−Q_3_). Categorical data are expressed as number (n) and percentage (%).

## Results

**Fig. 1:**
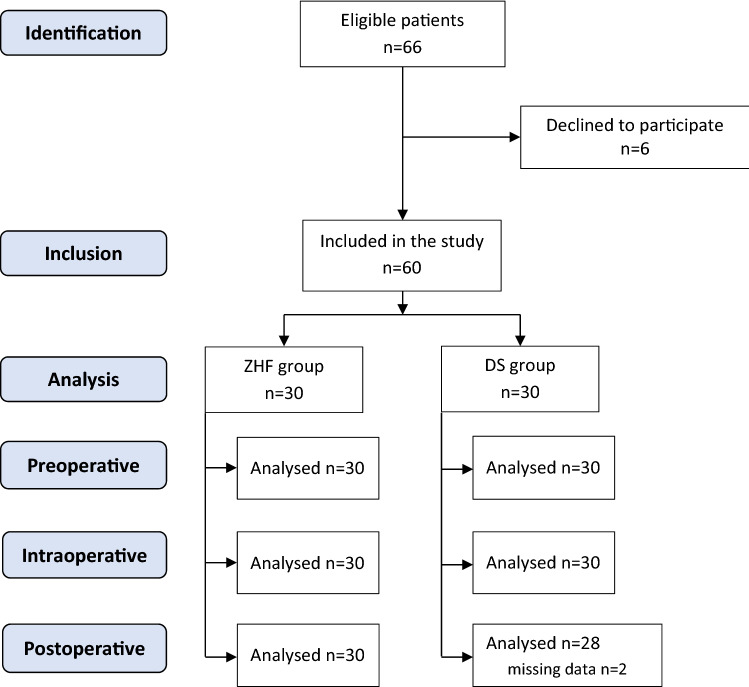
Flow diagram

Sixty patients were recruited to the study between May and November 2019. Of these, thirty patients were fitted with bilateral ZHF sensors (ZHF group), and thirty patients were fitted with both a ZHF sensor and a DS sensor (DS group). The postoperative data of two patients in the DS group were lost due to problems in data collection (Fig. 1). Patient characteristics and relevant perioperative data are presented in Table 1.

**Table 1:** Patient characteristics and relevant perioperative data

	Zero heat flux group		Double sensor group	
	n = 30		n = 30	
	n/median	%/Q_1_ − Q_3_	n/median	%/Q_1_ − Q_3_
Age (years)	71	62–74	69	64–73
BMI (kg/m^2^)	29	27–32	30	28–33
Female	19	63.3	16	53.3
ASA				
I	1	3.3	7	23.3
II	18	60.0	9	30.0
III	11	36.7	14	46.7
Warming				
EasyWarm	16	53.3	14	46.7
BairHugger	14	46.7	16	53.3
Side of surgery				
Right	17	56.7	17	56.7
Bicain spinal 5 mg/ml (ml)	1.6	1.5–2.0	1.5	1.4–1.6
Preoperative holding area temperature (°C)	20.7	20.5–20.9	21.5	20.9–21.8
Operating room temperature (°C)	18.6	17.9–18.9	19.2	18.9–19.9
Duration of prewarming (min)	30	30–32	32	30–42
Duration of surgery (min)	60	55–70	57	49–63
Propofol sedation intraoperatively	25	83.3	25	83.3
Propofol (mg/kg/h)	3.2	2.5–4.4	4.0	3.3–4.2
Hypothermia^a^ intraoperatively	17	56.7	11	36.7

*BMI* body mass index, *ASA* American Society for Anesthesiologists

^a^Core temperature value below 36.0 °C measured with zero heat flux sensor placed on the right side of the forehead

The ZHF and DS sensors were placed on the forehead before initiating preoperative warming. As the temperature value of the ZHF sensor stabilised within four minutes, the value of the DS sensor only stabilised after ten minutes. The unstable temperature data were excluded from the statistical analyses.

### Comparison between two ZHF sensors

**Fig. 2:**
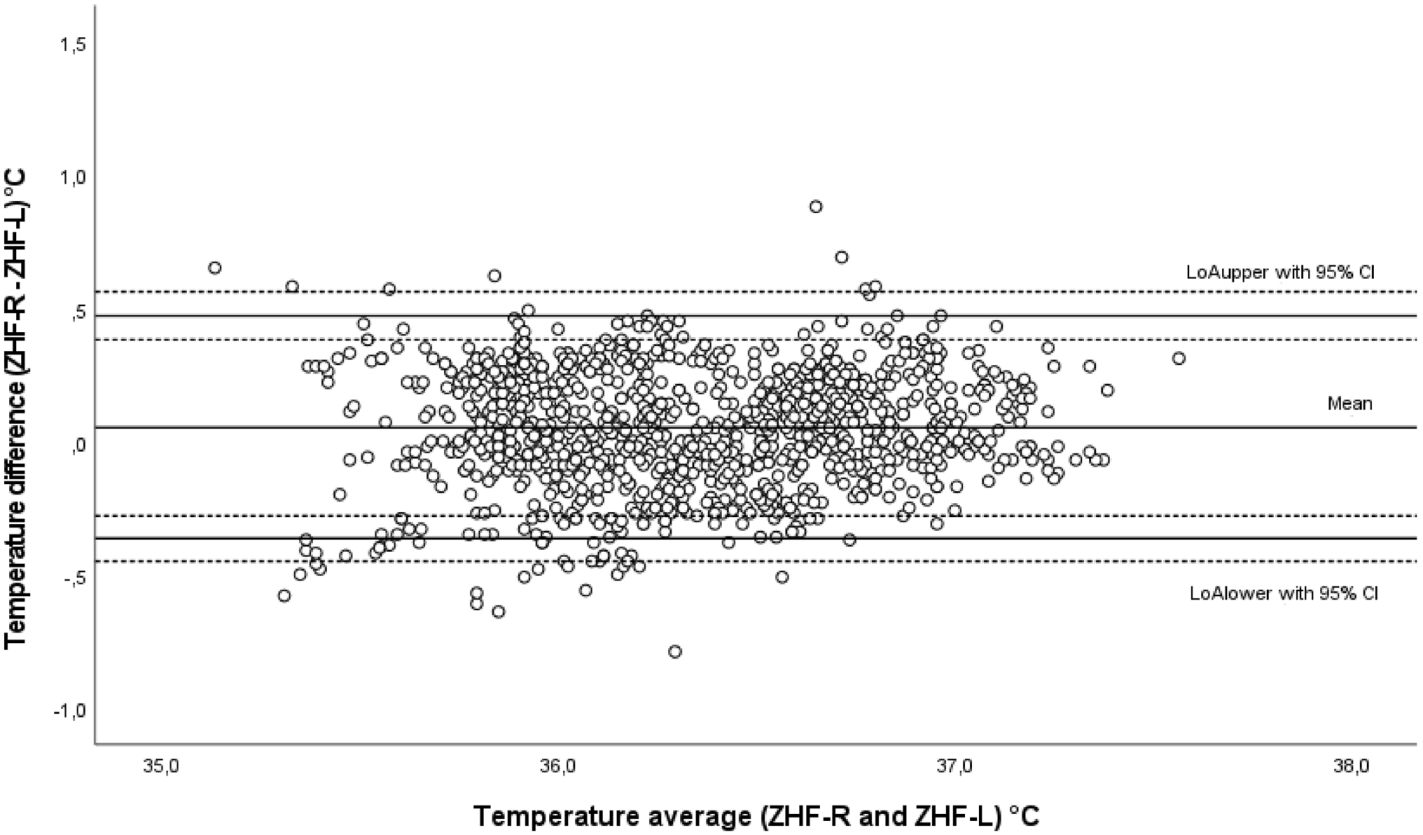
Bland–Altman plot of the ZHF group. Comparison of bilateral ZHF sensors. *ZHF* zero heat flux, *R* right forehead, *L* left forehead, *LoA* 95% limits of agreement, *CI* confidence interval

**Fig. 3:**
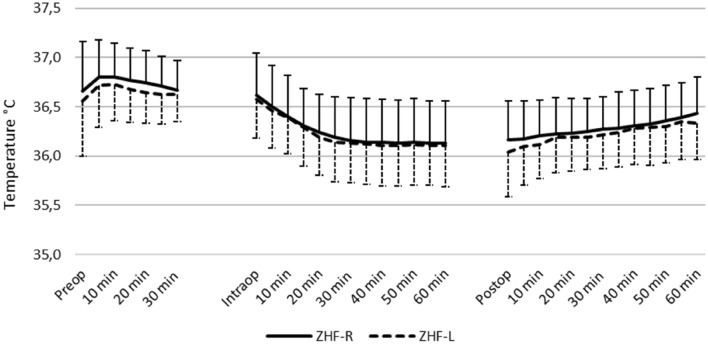
Chronological temperature changes of the right and left ZHF sensors. Mean with standard deviation. *ZHF* zero heat flux, *R* sensor placed on the right side of the forehead, *L* sensor placed on the left side of the forehead

A total of 1261 measurement pairs were obtained at 5-min intervals perioperatively (preoperative n = 332, intraoperative n = 512, postoperative n = 417). Results and BA plot are presented in Table 2 and in Fig. 2, respectively. Chronological changes of T_ZHF-R_ and T_ZHF-L_ are illustrated in Fig. 3.

**Table 2:** Results of the evaluation of the ZHF and DS methods

	Preoperative	Intraoperative	Postoperative	Overall
*ZHF group*				
Temperature ranges (°C)				
ZHF-R	35.5–37.7	35.0–37.3	35.5–37.2	35.0–37.7
ZHF-L	35.3–37.4	35.2–37.4	35.0–37.1	35.0–37.4
Mean difference ± 95% LoA with 95% CI (°C)	0.07 ± 0.37 with ± 0.08	0.04 ± 0.46 with ± 0.13	0.06 ± 0.39 with ± 0.10	0.05 ± 0.42 with ± 0.08
Proportion of temperature differences ≤ 0.5 °C (%)	99	98	99	99
LCCC (± 95% CI)	0.86 (± 0.03)	0.84 (± 0.03)	0.85 (± 0.03)	0.88 (± 0.01)
*DS group*				
Temperature ranges (°C)				
ZHF-R	36.1–37.4	35.4–37.3	35.3–37.1	35.3–37.4
DS	35.2–37.8	34.4–37.9	34.6–36.8	34.4–37.9
Mean difference ± 95% LoA with 95% CI (°C)	0.13 ± 0.80 with ± 0.20	0.36 ± 0.90 with ± 0.24	0.46 ± 0.76 with ± 0.22	0.33 ± 0.88 with ± 0.20
Proportion of temperature differences ≤ 0.5 °C (%)	82	61	61	66
LCCC (± 95% CI)	0.51 (± 0.06)	0.51 (± 0.06)	0.45 (± 0.05)	0.59 (± 0.03)

### Comparison between ZHF and DS methods

**Fig. 4:**
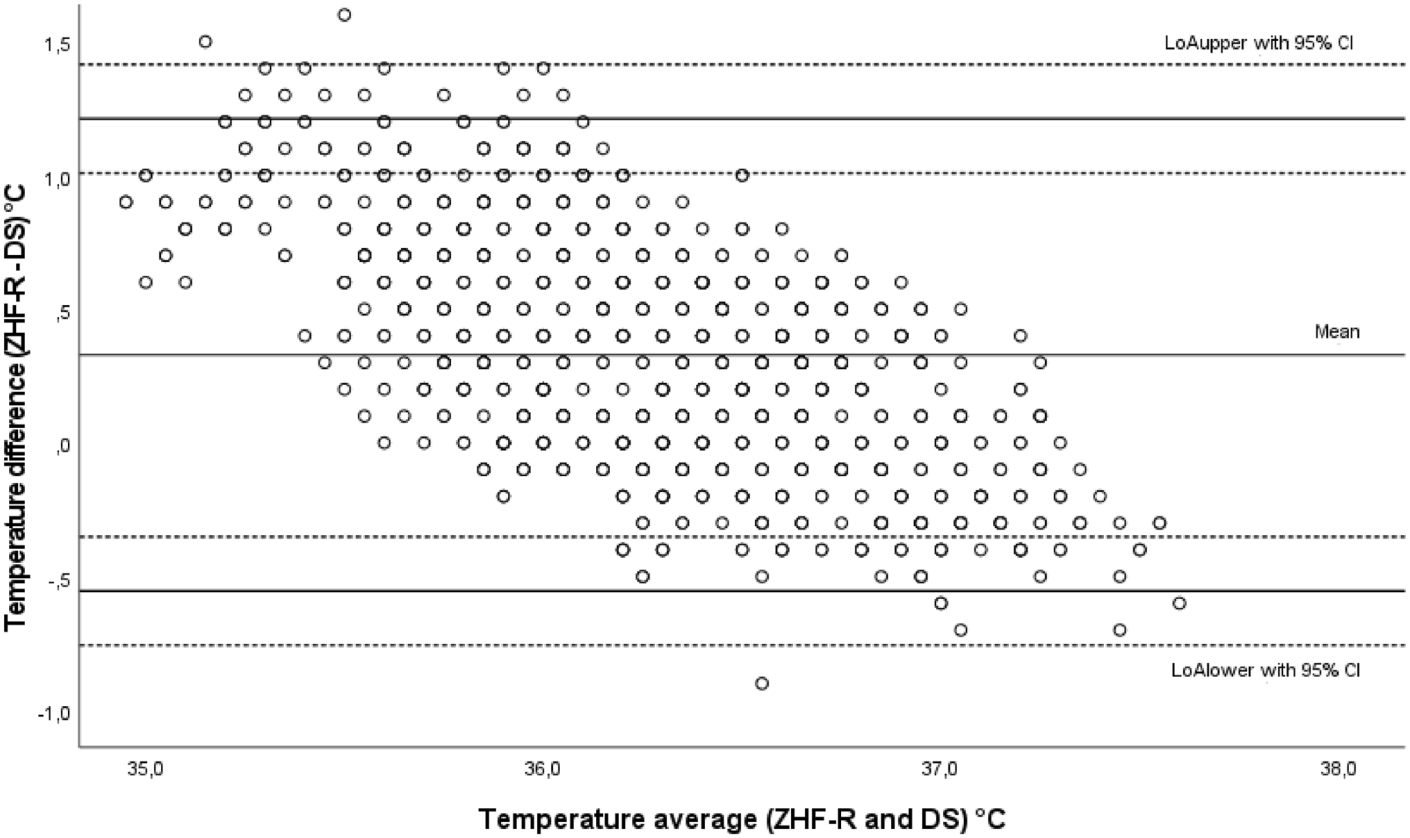
Bland–Altman plot of the DS group. Comparison of ZHF and DS temperature measurement methods. *ZHF* zero heat flux, *R* right forehead, *DS* double sensor, *LoA* 95% limits of agreement, *CI* confidence interval

**Fig. 5:**
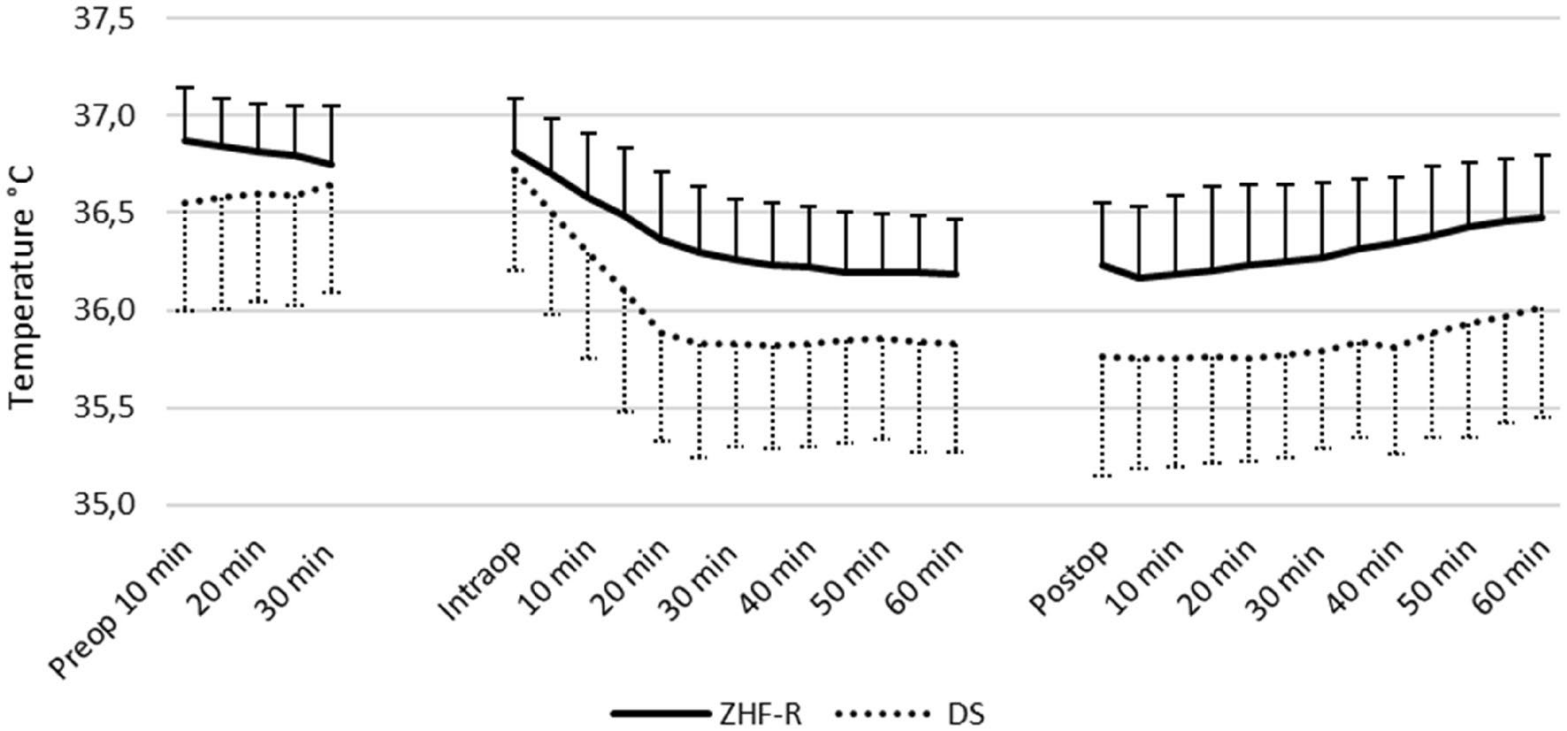
Chronological temperature changes of the ZHF-R and DS sensors. Mean with standard deviation. *ZHF-R* zero heat flux sensor placed on the right forehead, *DS* double sensor placed on the left forehead

A total of 1129 measurement pairs were obtained at 5-min intervals perioperatively (preoperative n = 301, intraoperative n = 484, postoperative n = 344). Results and BA plot are presented in Table 2 and in Fig. 4, respectively. Chronological changes of T_ZHF-R_ and T_DS_ are presented in Fig. 5.

## Discussion

The findings of this observational prospective study demonstrate that the side of the forehead does not influence the temperature reading of ZHF sensors. The mean difference between temperatures obtained by ZHF and DS sensors was bigger than that between two ZHF sensors. Further, the lower the core temperature was, the larger the mean difference between the ZHF and DS sensors.

The ZHF method has previously been evaluated in many clinical studies, whereas only a few clinical studies have compared the DS method to standard invasive temperature monitoring. The agreement of ZHF temperature with PA, oesophageal and nasopharyngeal temperatures has been shown to be sufficiently accurate [16–18, 28–30]. The DS method has been estimated to be comparable to oesophageal, bladder and femoro-iliac artery temperatures [20, 21, 31]. Although the mean difference between DS and PA temperatures in cardiac surgical patients was small, the 95% limits of agreement were over a degree [22, 23].

We are unaware of previous studies that have compared two simultaneous ZHF sensors in a single patient. Regardless of the identical method, the observed dispersion of temperature difference was small, but greater than we expected. There may be several patient- or sensor-related reasons for this greater than expected dispersion of temperature difference. First, changes in body and head position may have influenced cerebral blood flow [32], causing bilateral temperature values to diverge. Second, according to the manufacturer, the sensor reaches a depth of 1 to 2 cm below the skin [16]. Finally, the anatomical focus of the sensor cannot be determined, and therefore the temperature measurement point remains inexact [29], leading to measurement inaccuracy.

In general, the DS sensor yielded lower temperature values than the ZHF sensor throughout the study. Further, intraoperative temperature drop was greater with the DS sensor than with the ZHF sensor. The increased difference between the temperatures yielded by the DS sensor and the rectal temperature recordings at lower core temperatures has also been reported in volunteer studies by Gunga [33, 34]. In our study, the drop was seen in a cool ambient temperature, where many patients became hypothermic. In addition, we noticed that the size of the DS sensor sometimes hampers a perfect fit to the forehead skin and may therefore have contributed to a partial unfastening of the sensor, leading to unsatisfactory contact and even inaccurate readings. The possible partial insulation of the sensor may have allowed heat loss to influence the temperature recording and heat flux calculation, which results in a lower observed temperature. However, the anaesthesia management itself does not appear to have had an influence on the sensor feasibility, since the DS sensor has been shown to perform equally well in patients undergoing both regional and general anaesthesia [21].

The sufficient accuracy of a thermometer is considered an offset from the reference temperature of 0.5 °C, because normal circadian fluctuations are within this range [35]. Further, 0.5 °C is the smallest difference that has been shown to be associated with hypothermia-induced complications [36]. The mean difference with 95% LoAs remained within 0.5 °C between the ZHF sensors. The mean difference between the ZHF and DS sensors was 0.33 °C, which is acceptable, but the difference together with the observed 95% LoAs (i.e., 0.88 °C) exceeds the proposed limit of 0.5 °C and was therefore unsatisfactory.

Core temperature should be maintained over 36.0 °C perioperatively, and a patient with hypothermia should be actively warmed [37]. Such warming, though mandatory, increases costs and the workload of personnel, produces waste and may cause sedation and intubation-related risks when performed in the postanaesthetic care unit. For proper patient care, the appropriate monitoring system should be used but possible limitations of the measuring methods must be recognised. The incidence of hypothermia has been shown to vary from 11 to 60% in previous prospective observational studies performed in patients receiving NA for joint arthroplasty [38, 39]. In our study, the overall incidence of intraoperative hypothermia measured with T_ZHF-R,_ was 47%. Our results together with those of previous studies underline the need for the accurate and adequate non-invasive monitoring of core temperature.

The major limitation of our study was that we did not have a standard core temperature measurement site as a reference method. Hence, no conclusion on the superiority of either of these methods can be drawn based on these results. However, the agreement of the ZHF method with standard core temperature measurement methods has been shown to be precise and acceptable for clinical use [16, 17, 40]. The ZHF method was chosen as a reference because it is the primary core temperature measurement method used under NA in our hospital, and we could not predispose the conscious patients to invasive core temperature measurements.

The strengths of this study were that our study included not only intraoperative, but also pre- and postoperative temperature measurements with multiple observations per patient. Further, surgery and anaesthesia were similar for all patients, and the same ZHF control and DS adapter units were used throughout the study. Finally, we measured the temperature from both sides of the forehead to ensure that two different measurement methods, placed either side of the forehead, may be compared.

In future, the ZHF method should be evaluated in different patient populations undergoing neuraxial anaesthesia. In addition, more studies that compare the DS method with other non-invasive and standard temperature measurement methods under various circumstances are needed, as the existing clinical studies are scarce and appear to report conflicting results.

In conclusion, based on the findings of our study, the ZHF method has good internal validity, and the temperature reading is not dependent on the side of the forehead. The DS method shows lower temperature values than the ZHF method, especially when the core temperature is low. However, based on our results we do not know which one of the two methods measures core temperature more accurately.
